# Whole genome sequencing in *Drosophila virilis* identifies *Polyphemus*, a recently activated Tc1-like transposon with a possible role in hybrid dysgenesis

**DOI:** 10.1186/1759-8753-5-6

**Published:** 2014-02-20

**Authors:** Justin P Blumenstiel

**Affiliations:** 1 Department of Ecology and Evolutionary Biology, University of Kansas, 1200 Sunnyside Avenue, Lawrence KS 66049, USA

**Keywords:** Hybrid dysgenesis, *Drosophila virilis*, Transposable element, *Penelope*, piRNA, Genome instability, Epigenetics

## Abstract

**Background:**

Hybrid dysgenic syndromes in *Drosophila* have been critical for characterizing host mechanisms of transposable element (TE) regulation. This is because a common feature of hybrid dysgenesis is germline TE mobilization that occurs when paternally inherited TEs are not matched with a maternal pool of silencing RNAs that maintain transgenerational TE control. In the face of this imbalance TEs become activated in the germline and can cause F1 sterility. The syndrome of hybrid dysgenesis in *Drosophila virilis* was the first to show that the mobilization of one dominant TE, the *Penelope* retrotransposon, may lead to the mobilization of other unrelated elements. However, it is not known how many different elements contribute and no exhaustive search has been performed to identify additional ones. To identify additional TEs that may contribute to hybrid dysgenesis in *Drosophila virilis*, I analyzed repeat content in genome sequences of inducer and non-inducer lines.

**Results:**

Here I describe *Polyphemus*, a novel Tc1-like DNA transposon, which is abundant in the inducer strain of *D. virilis* but highly degraded in the non-inducer strain. *Polyphemus* expression is also increased in the germline of progeny of the dysgenic cross relative to reciprocal progeny. Interestingly, like the *Penelope* element, it has experienced recent re-activation within the *D. virilis* lineage.

**Conclusions:**

Here I present the results of a comprehensive search to identify additional factors that may cause hybrid dysgenesis in *D. virilis. Polyphemus*, a novel Tc1-like DNA transposon, has recently become re-activated in *Drosophila virilis* and likely contributes to the hybrid dysgenesis syndrome. It has been previously shown that the *Penelope* element has also been re-activated in the inducer strain. This suggests that TE co-reactivation within species may synergistically contribute to syndromes of hybrid dysgenesis.

## Background

Hybrid dysgenesis, a syndrome of sterility and increased mutation in crosses between different strains of the same species, was first shown in *Drosophila melanogaster* to be driven by *P* elements that are inherited paternally, but not maternally [1-4]. Activation of this DNA transposon subsequently leads to germline DNA damage and sterility [5-8]. In *D. melanogaster*, transposable element (TE) mediated syndromes of hybrid dysgenesis are also driven by the *I* element retrotransposon [9] and the *hobo* DNA transposon [10]. An important syndrome of hybrid dysgenesis has also been characterized in *D. virilis*[11-14]. This syndrome is significant as it is accompanied by mobilization of different, unrelated transposable element families [13,15]. Critically, even elements such as the *Ulysses* retrotransposon that are evenly distributed between strains become mobilized in this cross. Recent studies using genome sequencing approaches indicate that *P* elements also induce the mobilization of other elements in *D. melanogaster*[5]. Thus, co-mobilization of TEs may be a common feature of dysgenic syndromes but the mechanism by which TE co-mobilization occurs is poorly understood. One mechanism that has been proposed is that DNA damage caused by the activation of one TE family disrupts piRNA silencing mechanisms in the germline *via* the DNA damage response [5]. In this model, disrupted piRNA silencing in turn leads to activation of normally repressed and unrelated TEs. Alternatively, DNA damage arising from transposition may drive activation of TEs through other mechanisms. Additionally, it has been proposed that, like viruses, TEs may encode suppressors of RNA silencing [16]. In this case, the expression of a suppressor of RNA silencing encoded by a single activated TE could lead to global TE de-repression. Finally, it is important to consider the possibility that multiple TE families may be more abundant within inducer strains [17]. Thus, the activation of multiple TE families in a dysgenic syndrome may also be explained by independent mechanisms acting across each family.

To distinguish among these hypotheses, it is critical to define the landscape of TE copy number imbalance between inducer and non-inducer strains in hybrid dysgenic syndromes. Many previous studies indicate that the *Penelope* element is likely to be the main driver of hybrid dysgenesis in *Drosophila virilis*. It is the only known element with multiple, active copies in the inducer strain and for which active copies are entirely absent from the non-inducer strain [11,17,18]. In addition, its expression is greatly increased in the gonads of dysgenic progeny and injection of embryos with *Penelope* constructs can lead to increased incidence of TE mediated mutation [11,14,19]. However, additional studies indicate that while *Penelope* may be the dominant cause of sterility, other factors may also contribute. For one, some strains of *D. virilis* that behave as neutral strains - maternally protecting against dysgenesis but not inducing it paternally - lack piRNAs from the *Penelope* element in their ovaries [20]. If *Penelope* is the sole cause of dysgenesis, it is difficult to explain how these strains protect against the induction of dysgenesis since mothers would be unable to provide *Penelope* piRNA to the next generation. Second, two additional TEs - *Helena* and *Paris* - also show high abundance of active, euchromatic copies in the inducer strain and lower abundance in the non-inducer strain. Evidence suggests these two elements also contribute to the sterility phenotype [17]. Whether these three elements act synergistically to cause sterility is not known. It is also not known whether they jointly contribute to the mobilization of other elements such as *Ulysses*.

The discovery of these candidate inducer elements - *Penelope*, *Helena*, and *Paris* - was facilitated by the recovery of TE insertions that gave rise to visible mutations during dysgenic co-mobilization. Thus, it has not been clear whether additional elements may contribute to the dysgenic syndrome in *D. virilis*. Here, I present the first systematic effort to identify additional TEs that may cause hybrid dysgenesis in *D. virilis*.

## Results and discussion

**Figure 1:**
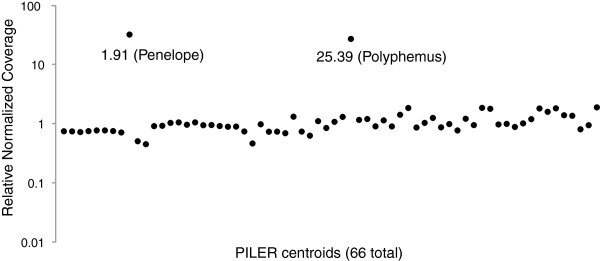
**Relative mapping abundance for all 66 PILER centroids from genome sequence reads (100 bp reads) of strains 160 and 9.** Mapped reads were normalized to all reads mapped to the reference genome using BWA-MEM. Two PILER centroids show high abundance in strain 160: 1.91, which corresponds to the *Penelope* element, and 25.39, which corresponds to *Polyphemus*.

To identify additional TEs that may contribute to the hybrid dysgenesis syndrome of *D. virilis*, I performed whole genome, 100 bp paired-end Illumina sequencing of DNA collected from inducer (strain 160) and non-inducer (strain 9) flies. Based on a genome size of 364 Mb estimated from flow cytometry [21,22], sequencing yielded approximately 24 X and approximately 21 X coverage for strain 160 and strain 9, respectively. After trimming for quality (https://github.com/najoshi/sickle), reads (one single end from each pair) were then mapped using BWA-MEM [23] to a library of *D. virilis* repeat sequences computationally predicted by the PILER program [24]. Figure 1 indicates the ratio for the number of reads (160:9) mapping to each PILER centroid, normalized by total number of reads mapped to the reference genome. From this, I identified centroid.25.39 to be enriched about 27-fold in strain 160 relative to strain 9 (*P* <0.001, chi-squared test), in a ratio similar to that observed with the centroid corresponding to the *Penelope* element (about 32-fold; Figure 1). This suggested that centroid.25.39 may correspond to an element that, like *Penelope*, is in excess in the inducer strain. Centroid.25.39 was therefore further characterized.

**Figure 2:**
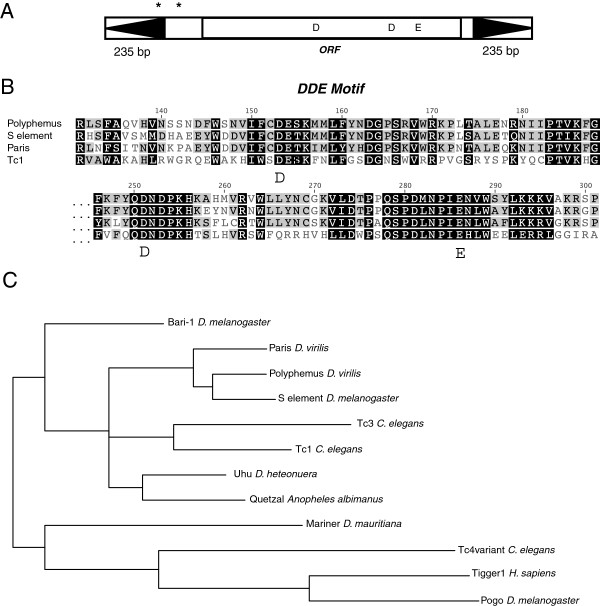
***Polyphemus *is a cut and paste transposon belonging to the Tc1 family. (A)** Overall structure of *Polyphemus* with 235-bp inverted repeats indicated by black arrows. Asterisks indicate putative alternate translation start sites. Position of the DDE motif that catalyzes the transposition reaction is indicated. **(B)** Alignment of amino acids that contain the DDE motif from closely related members of the Tc1 family: the founding Tc1 as well as the *S* element (from *D. melanogaster*) and *Paris* (from *D. virilis*). **(C)** Phylogeny of the Tc1/mariner family. *Polyphemus*, *Paris*, and *S* element form a clade within the Tc1 group.

To determine the consensus repeat sequence corresponding to centroid.25.39, I performed blastn with this computationally predicted repeat against the *Drosophila virilis* reference genome (the reference strain also induces hybrid dysgenesis). After performing several rounds of iterated blast, I identified and extracted the consensus sequence of a highly repeated Tc1-like transposon with 235-bp inverted flanking repeats that I have designated *Polyphemus* (Figure 2A and Additional file 1). Within this sequence there is an open reading frame that corresponds to a 344 amino-acid sequence with 65% identity (beginning to end) to the *S* element previously identified in *D. melanogaster*[25] and 59% identity (beginning to end) to the *Paris* element identified in *D. virilis*[15]. Both of these elements belong to the Tc1/*mariner* superfamily of cut-and-paste DNA transposons. Conservation of the catalytic DDE domain is noted in the comparison to the Tc1 element (Figure 2B). Phylogenetic analysis indicates that *Polyphemus* is located within the Tc1 clade of the larger Tc1/*mariner* group of DNA transposons (Figure 2C). Furthermore, it is most closely related to the *S* element and *Paris*. Interestingly, there are two alternate translation start codons that extend the putative reading frame up to 57 codons and into the first inverted repeat. However, the extended 57 amino acid sequence shares no sequence similarity to any known protein and therefore the transcription start site is likely downstream of these alternate translation start sites.

**Figure 3:**
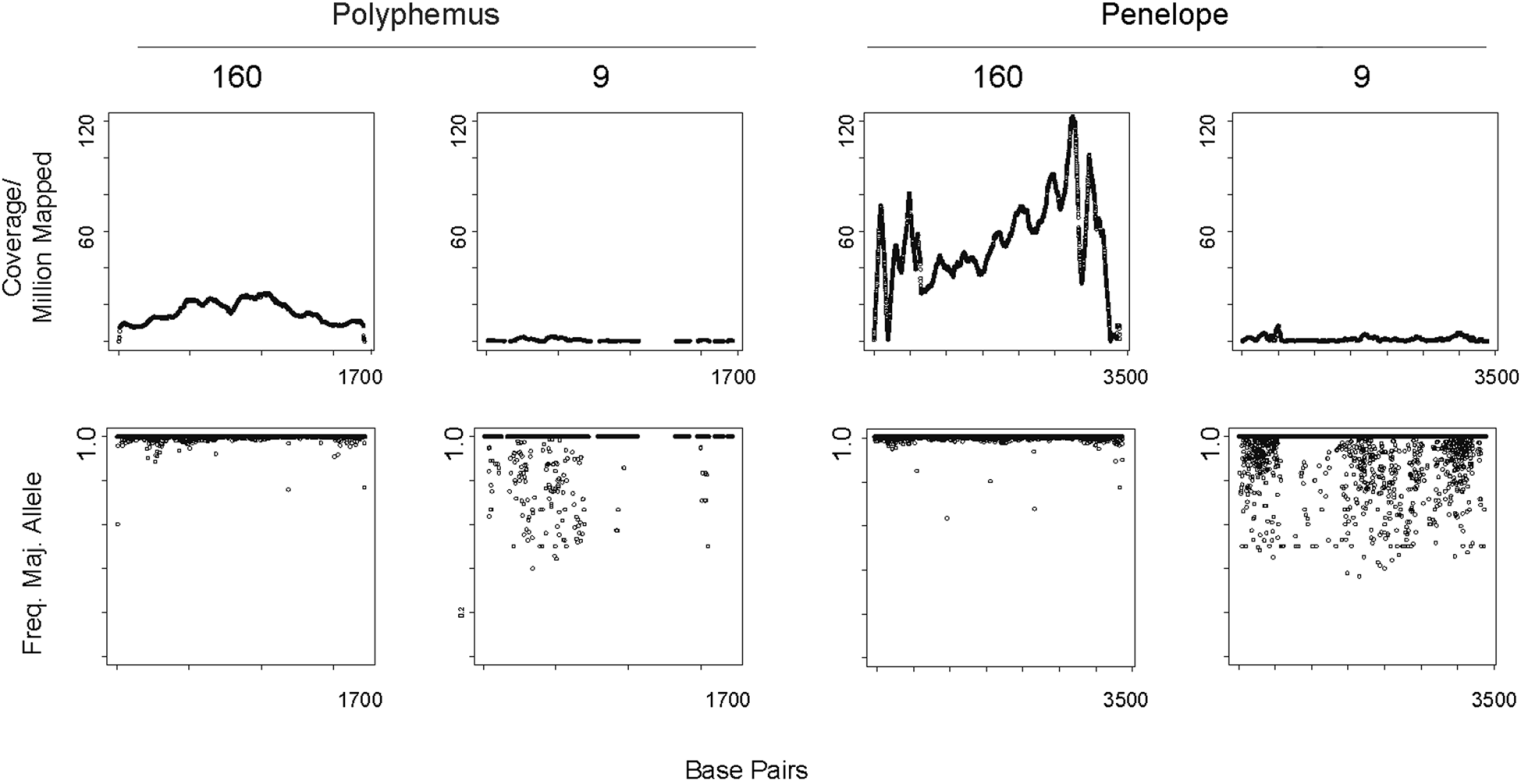
**Mapping coverage and sequence heterogeneity for *Polyphemus *and *Penelope *from strain 160 and strain 9 genomic reads.** For all plots, mapping coverage and sequence heterogeneity is shown along the length of the element. For *Polyphemus*, this is 1,704 bp. For *Penelope*, this is 3,394 bp. Read mapping coverage is measured on a per nucleotide basis, normalized by 1 million mapped reads. Based on coverage/million reads mapped, both *Polyphemus* and *Penelope* are enriched in strain 160. *Penelope* shows greater excess than *Polyphemus*. Using piledriver, I also determined sequence heterogeneity among mapped reads for *Polyphemus* and *Penelope* by scoring the frequency of the most common variant at each nucleotide position. In strain 160, *Polyphemus* and *Penelope* mapped reads are highly similar. In strain 9, mapped reads show great heterogeneity.

Based on coverage across the entire length of this element, representation of *Polyphemus* is greater in strain 160 than strain 9 (Figure 3). A similar analysis for the *Penelope* element confirms an even greater difference between strain 160 and strain 9. I next sought to determine sequence heterogeneity within the mapped reads since high sequence similarity among copies is often indicative of recent activity. To determine this, I extracted element specific mappings and used piledriver (https://github.com/arq5x/piledriver) to analyze sequence heterogeneity by counting the frequency, at each nucleotide position, of the most common variant (Figure 3). In strain 160, *Polyphemus* shows very little heterogeneity among mapped reads, suggesting recent activity of a single lineage. In contrast, there is great heterogeneity among mapped reads for *Polyphemus* in strain 9. Similar results are also observed for *Penelope*, for which strain 9 is known to only have degraded, non-functional copies [26,27].

**Figure 4:**
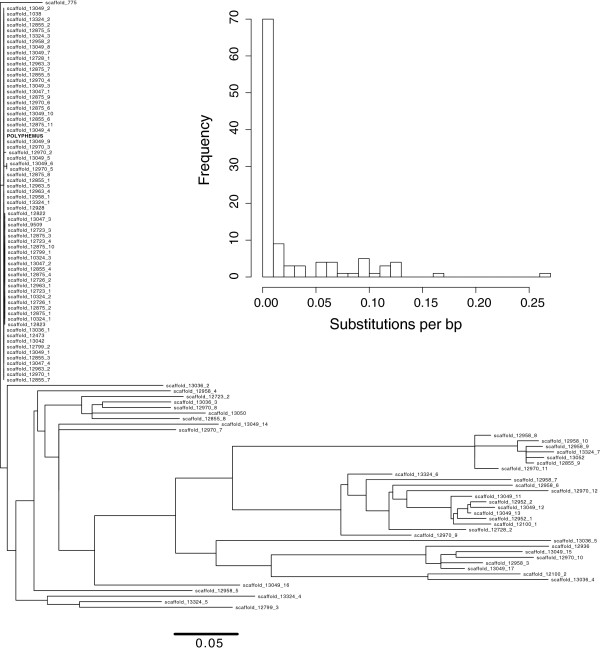
**Phylogenetic analysis of *Polyphemus *fragments identified by blastn from the reference *D. virilis *genome.** Phylogenetic tree was generated among all aligned fragments using GARLI. Distribution of branch lengths, showing a broad distribution of older fragments, is shown within the inset, indicating an older time of activity centered around 0.10 subs/bp. Note: since fragments were used, not all represent full length elements.

Using available genome assemblies, I then investigated the presence of this element across all available arthropod genomes to determine if it may have been recently derived from another known species, analogous to the way the *P* element in *D. melanogaster* was derived from *D. willistoni.* Using blastn with default match and mismatch scores (Match: 1, Mismatch: -3, Gap Open: 5, Gap Extension: 2) no hits were identified with an E-value cutoff of less than E-10 in any other species. Thus, it is unlikely to have entered *D. virilis* via recent horizontal transfer from any of these species with sequenced genomes. Using blastn solely on the available *D. virilis* reference genome I found that, in addition to the many nearly identical copies, many fragments were identified with E-values ranging from E-40 to E-180. Thus, while no hits with similar levels of significance were found outside *D. virilis*, a wide range of divergent fragments were identified within *D. virilis*. This suggests that lineages of *Polyphemus* have been residing within the *D. virilis* lineage for a significant period of time. Therefore, I investigated the evolutionary history of *Polyphemus* within the *Drosophila virilis* genome by generating a phylogenetic tree of all *Polyphemus* fragments (coding sequence only) in the assembled *Drosophila virilis* genome using GARLI [28] with a GTR model and no rate heterogeneity with empirical base frequencies. From the phylogenetic analysis (Figure 4), it is apparent that there is an active clade that has recently proliferated on a background of highly divergent fragments. Considering the distribution of terminal branch lengths, many are around 0.10 substitutions per base pair long. Assuming a per nucleotide substitution rate of 1.45 × 10^-9/bp/gen [29] and 10 generations per year, many of these fragments are about 7 million years old. Thus, it appears that at least one *Polyphemus* lineage has resided in the *D. virilis* genome for a long time and has become recently activated within the species, including lines that induce hybrid dysgenesis. A similar pattern has previously been demonstrated for the *Penelope* element [26]. One possibility is that *Polyphemus* re-invaded *D. virilis* via horizonal transfer from another member of the *D. virilis* group.

**Figure 5:**
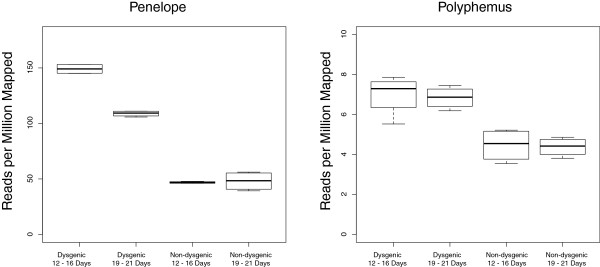
**Expression of *Penelope *and *Polyphemus *measured in RNA-seq reads per million mapped.** RNA was collected from 0 to 2-hour-old embryos of F1 females (at 12 to 16 days and 19 to 21 days) from both directions of the cross. Error indicates error derived from technical replicates (Barcode and Lane effects).

To investigate whether *Polyphemus*, like *Penelope*, shows increased expression when inherited paternally but not maternally, I analyzed RNA-seq data from 0 to 2-hour-old embryos laid by reciprocal F1 females of the dysgenic and non-dysgenic crosses. The sterility phenotype of dysgenesis is not fully penetrant and these embryos from this direction of the cross are therefore derived from F1 females that escape sterility. F1 females of two different ages were used to examine the dynamics of expression over lifetime and embryos rather than ovaries were used to avoid problems associated with measuring gene expression in dysgenic ovaries that may be skewed in representation of somatic and germline material. Being 0 to 2 hours old, these embryos provide a measure of strictly germline expression in the F1 female. As has been previously demonstrated, *Penelope* expression is significantly higher in the germline of females from the dysgenic direction of the cross (Figure 5). Interestingly, this difference depends on the age of the F1 female (Table 1). *Penelope* germline expression is decreased in older F1 females from the dysgenic direction of the cross. *Polyphemus* expression is also higher in the germline of F1 females from the dysgenic cross, though the level of expression and magnitude of difference is smaller compared to *Penelope* (Figure 5). Interestingly, this effect does not depend on the age of the F1 female (Table 2). Thus, like *Penelope*, *Polyphemus* shows increased expression when inherited paternally.

**Table 1:** ***Penelope*** **RNA-seq analysis: ANOVA results**

	**Df**	**Sum Sq**	**Mean Sq**	**F**	**Pr (>F)**
Treat	1	26600	26600	1594.6615	2.32E-12
Age	1	1523.7	1523.7	91.3473	2.40E-06
Barcode	2	137	68.5	4.1071	0.04988
Treat X age	1	1722.3	1722.3	103.2484	1.37E-06
Residuals	10	166.8	16.7		

Results were from two treatments (dysgenic and non-dysgenic), which were each collected from two different ages (12 to 16 days old and 19 to 21 days old). For each of these four samples, two different RNA-seq libraries were constructed with different barcodes and libraries were run in duplicate.

**Table 2:** ***Polyphemus*** **RNA-seq analysis: ANOVA results**

	**Df**	**Sum Sq**	**Mean Sq**	**F**	**Pr (>F)**
Treat	1	24.9251	24.9251	43.4233	6.16E-05
Age	1	0.0564	0.0564	0.0983	0.7604
Barcode	2	0.9773	0.4886	0.8513	0.4556
Treat X age	1	0.0039	0.0039	0.0068	0.9359
Residuals	10	5.74	0.574		

Results were from two treatments (dysgenic and non-dysgenic), which were each collected from two different ages (12 to 16 days old and 19 to 21 days old). For each of these four samples, two different RNA-seq libraries were constructed with different barcodes and libraries were run in duplicate.

## Conclusions

Here I describe *Polyphemus*, a new Tc1-like transposable element in *D. virilis* that may contribute to the hybrid dysgenic syndrome*.* Whereas highly similar copies are abundant in the inducer strain, only degraded copies are found in the non-inducer strain. Nonetheless, the lack of active copies in the non-inducer strain does not suggest an entirely new invasion of *Polyphemus* into *D. virilis*. Instead, phylogenetic analysis indicates that different lineages of *Polyphemus* have persisted in *D. virilis* for many years. Against this history, it appears that a *Polyphemus* variant has now become re-activated. This re-activation may have occurred via a horizontal transfer event from another member of the *D. virilis* group that has maintained an active *Polyphemus* lineage since divergence from *D. virilis*. Alternatively, an active lineage of *Polyphemus* may have continuously persisted in *D. virilis*. In this case, individuals within the species would have been segregating with respect to rare active copies of *Polyphemus*. In this scenario, strains or populations that maintain rare active copies may have functioned as reservoirs for later re-activation of *Polyphemus.* Interestingly, the *Penelope* element shows a similar pattern. Active copies of *Penelope* are highly abundant in the inducer strain, but this is not because *Penelope* is entirely new to the *D. virilis* lineage. The non-inducer strain 9 also possesses old, inactive copies and different variant lineages of *Penelope* are seen in different species of the *D. virilis* group. Thus, it appears that both of these elements have become re-activated from within the *D. virilis* group and now may jointly cause hybrid dysgenesis.

In *D. melanogaster*, three different element families are known to cause hybrid dysgenesis. In the most well understood P-M system, the *P* element invaded via horizontal from the distant *D. willistoni* species [30]. However, for the *I-R* system and the *hobo* system, both *I* elements [31] and *hobo* elements [32] have remnant copies residing in the *D. melanogaster* genome. This suggests that dysgenic syndromes may frequently result from re-activation of TE lineages that, in contrast to the *P* elements, have been long-term genomic residents. Interestingly, the presence of multiple different elements, such as *Paris* and *Helena*, that are also in excess in the inducer strain of *D. virilis* seems to indicate that TE control has been diminished in strain 160. Perhaps this has occurred by the same mechanism that leads to TE co-mobilization. If so, *Penelope* might be the cause of this TE excess in strain 160, but might not be the sole proximate cause of sterility in hybrid dysgenesis. For this reason, the mechanisms that are responsible for TE co-mobilization in dysgenic syndromes may also be relevant to understanding global TE dynamics within species after one or more TEs becomes re-activated.

Expression of *Polyphemus* is higher in the germline of dysgenic progeny, though not to the same magnitude as *Penelope*. In light of this, it is important to note that the sterility syndrome is evident early in development [33], not at the time that gonadal expression is typically measured. For this reason, TE expression in adult females that have escaped sterility - a necessary condition for measuring germline gene expression - may not be a perfect proxy for understanding TE expression early in development. The change in *Penelope* expression during the aging process indicates that TE activity is likely to be dynamic in the life of a dysgenic F1. For this reason, it will be critical to determine the patterns of germline activity for all four TEs early in development. Combined with genetic approaches, this may elucidate the causal factors of sterility and TE co-mobilization in the hybrid dysgenic syndrome of *Drosophila virilis.*

## Methods

### Genome sequencing and analysis of Polyphemus

DNA was collected from wandering third instar larvae from strain 160 (the inducer strain) and strain 9 (the non-inducer strain). DNA was then sonicated and fragments between 400 to 500 bp were selected for Illumina library preparation. Each library was 100 bp, paired-end sequenced on an individual lane of a GAII using, yielding 43.7 million (strain 160) and 37.6 million (strain 9) read pairs. Since pairs are not independent samples, only single ends of each pair were selected for this analysis. Single reads were quality trimmed using the Sickle application with default settings. Subsequent to quality trimming, reads were mapped using BWA-MEM to a pre-computed PILER library of repeat sequences from *D. virilis* (ftp://ftp.flybase.net/genomes/aaa/transposable_elements/PILER-DF). Total read counts for each centroid were normalized to the total number of reads mapping back to the reference and the ratio of 160:9 normalized reads was determined. From this, the 1720 bp PILER centroid.25.39 was identified as highly abundant in strain 160 but not strain 9. This centroid was used in a blastn search of the *D. virilis* reference genome and many nearly exact copies were identified. Three of these elements were extracted with 1000 bp of flanking sequence from each side. Reciprocal pairwise blast between these three fragments identified a core sequence 1,708 bp long of near identify among these fragments. Further annotation of this sequence was performed using Geneious. Phylogenetic analysis of Tc1/*mariner* members was performed using MrBayes on a MUSCLE amino acid alignment until the average standard deviation of split frequencies was less than 0.01. Analysis of heterogeneity among fragments was performed using piledriver to examine sequence heterogeneity of reads mapping to the respective TE. From the piledriver output, the frequency of the most common base at each position was calculated. Phylogenetic analysis was performed by collecting all blastn fragments from the *D. virilis* reference genome (excluding inverted repeats) with E values better than E-5. Fragments smaller than 100 bp were removed from the blast output and the blast output anchored to the active sequence was used as an alignment. Tree searching was performed using GARLI on the CIPRES server (http://www.phylo.org/) with a GTR model, no rate heterogeneity and empirical base frequencies. Terminal branch length distributions were extracted from the resulting tree file.

### Expression analysis

RNA was collected from 0 to 2-hour-old embryos that were laid by F1 females of the dysgenic (strain 9 mothers and strain 160 fathers) and non-dysgenic (strain 160 mothers and strain 9 fathers) crosses. Embryos of F1 females were chosen to avoid the confounding effects that are presented by ovaries that may differ in somatic *vs.* germline tissue representation in dysgenic crosses. Dysgenic and non-dysgenic crosses were set up *en masse* and hundreds of F1 females were collected soon after eclosion. Hundreds of reciprocal F1 males from the same crosses were also collected, combined in equal proportions, then reallocated equally to the collected dysgenic and non-dysgenic F1 females in mating cages. This was done to ensure a sufficient egg lay from the F1 females escapers of the dysgenic cross. Such females, if only provided their dysgenic brothers, lay few eggs. Providing equivalent but mixed populations of reciprocal males to the female pools also ensures proper genetic control over paternal effects *en masse* since reciprocal males of the dysgenic and non-dysgenic crosses are genetically different. From these cages, eggs were collected over 0 to 2-hour egg lay durations and each collection was immediately flash frozen in liquid nitrogen. Collections were pooled into two different age classes based on the age of the mother: 12 to 16 days old (about 1 week after sexual maturity) and 19 to 21 days old.

Pooled RNA was collected and Illumina libraries were generated for single-end, 50 bp RNA-seq. To control for index effects each RNA sample was used to generate two index libraries for a total of eight libraries, each of which were run in replicate on two different lanes. Trimming and filtering was performed using the Galaxy server (https://usegalaxy.org/). Up to 16 bp were quality trimmed from the 3' end from each read and remaining reads with more than 2 bp with quality less than 20 were removed. Trimmed and filtered reads were mapped to TE sequences using CLC with mismatch, insertion and deletion scores equal to 2.3 and 3, respectively. Expression levels were measured by the number of reads that mapped each TE normalized by the total number of reads mapping to the reference genome. ANOVA was performed in R using the aov command and a model that included the effects of treatment (dysgenic *vs.* non-dysgenic), age, index, and an interaction between age and treatment.

## Abbreviations

GTR: Generalised time reversible; TE: Transposable element.

## Competing interests

The author declares that he has no competing interest.

## Supplementary Material

Additional file 1 **Sequence and Annotation of *Polyphemus.***
Click here for file
