# Supplementary material for: Whole genome sequencing in Drosophila virilis identifies Polyphemus, a recently activated Tc1-like transposon with a possible role in hybrid dysgenesis
Source: Mob DNA. 2014 Feb 20;5:6. doi: 10.1186/1759-8753-5-6 (PMC3941972; doi:10.1186/1759-8753-5-6)
Supplement: Additional file 1 — Sequence and Annotation of Polyphemus. [file 1759-8753-5-6-S1.doc]

Supplemental 1. Sequence and annotation of *Polyphemus.*

5-238: Inverted Repeat

1469-1704:Inverted Repeat

384-1418: ORF with S-Element Homology: Starts at methionine corresponding to start of S element ORF

213-1418: Alternate ORF - long: Translation Start Includes Inverted Repeat

294-1418: Alternate ORF - short: Translation Start Doesn't Include Inverted Repeat

>Polyphemus

ATACAGTATGTCAATAAAGTGTTTTTACTAACAAAAAAAAACTAATTTTTTGGGTTTAGTTGAAAATAAATGTACCTAAAAGTGAGAATTTTTTTTCAATTATAATTTATTTCGATTCTATATTTCTCCTAGAACTTAATGGCAAAAAGAATTGTTAAATAACATTACCCAAACATTTTATAAAATTAAAAAACTAAAAACATTCACATTTTATGCTGTCAATAAAGTGTTTTTACAGCTACGGTAAATTGTTTTGCCGTTCTCTTTTGGGTGATCCCGTTAGATTTGGGCCCATGCGTTTTGGCTTCTCTTGTGTCTTCGACTTTTCTTGTCATTTTCTATTGCAATTTATAACGTTTTGCTTTAATTTTATGAAAAATAAA**A**TGCCAGGAAAAAGAACAACTTTTGAAGTCGCTCAGCTGGTGTATTATAACCATCAGATGGGACGTCAAGTCTCGGAACTGGCTGATATGTTTAATTTATCAAAAGCAACGATATACAACATATTAAACAGAGCCAATAAGGAAGATAGGCTAGAGGCAAAGCCAGTATGTGGTCGACCCTGCAAAATTTCTGACAGAGATAAGCGAAAAATTCTGAGAAAAATTGAGAAAAATCCACAAATTTCGCTTAGGGATATTGCTCAGGAGTTCAAGGAAGAAAGTGGTGTTGATGTGTCACATGAAACTGTCCGAAAATTACTGAATTCCAATGACTACACATCACGAGTTGCCAGGAAAAAACCTCTACTATCGGCGGCGAATATTTTGAAGCGGCTATCGTTCGCTCAAGTCCATGTAAATAGTTCGAACGATTTTTGGAGTAACGTCATATTCTGTGACGAAAGCAAGATGATGCTATTCTACAACGATGGGCCATCCAGAGTCTGGAGAAAGCCACTAACAGCGTTGGAAAACCGCAATATTATTCCAACCGTTAAATTCGGAAAACTATCAGTGATGGTGTGGGGGTGTATTTCGAGCAAGGGTGTAGGAGATTTGACCTTCATTGAGAATACCATGGATGCTAGACAATATCTGAGCATTTTACAGACACATCTCGTCAGTAGTGCACAGAAATTTGGATTCTATGAGGACAATAAGCCAATTTTTAAATTTTACCAGGACAATGACCCGAAGCATAAAGCTCATATGGTAAGAGTTTGGCTGTTGTACAACTGTGGAAAGGTGTTAGACACTCCACCACAAAGTCCAGATATGAATCCAATAGAAAATGTGTGGTCATATTTGAAAAAAAAAGTCGCAAAGCGGAGTCCAAAATCAAAAACTGATTTAAAAGCTGCTGTACTGGAAGAATGGCAGAAGATTCCAGAAACATACATACAAAACCTTATTGTTTCAATGAAACGGCGATTGCAGGCTGTATGTGACGCTAATGGCAGCCACACAAAGTAT**TAA**AAAAAAATATTCCTGTTTTTTTAACATTGTTATTTAGTAATAAAGTTTCGTGTAAAAACACTTTATTGACAGCATAAAATTTGAATGTTTTTAGTTTTTTAATTTTATAAAATGTTTGGGTAATGTTATTTAACAATTCTTTTTGCCATTAAGTTCTAGGAAAAATATAGAATCGAAATAAATTAGAATTGAAAAAAAATTCTCACTTTTAGGTACATTTATTTTCAACTAAACCCAAAAAATTAGTTTTTTTTCGTTAGTCAAAACACTTTATTGACATACTGTACATA

>ORFwithSHomology

MPGKRTTFEVAQLVYYNHQMGRQVSELADMFNLSKATIYNILNRANKEDRLEAKPVCGRPCKISDRDKRKILRKIEKNPQISLRDIAQEFKEESGVDVSHETVRKLLNSNDYTSRVARKKPLLSAANILKRLSFAQVHVNSSNDFWSNVIFCDESKMMLFYNDGPSRVWRKPLTALENRNIIPTVKFGKLSVMVWGCISSKGVGDLTFIENTMDARQYLSILQTHLVSSAQKFGFYEDNKPIFKFYQDNDPKHKAHMVRVWLLYNCGKVLDTPPQSPDMNPIENVWSYLKKKVAKRSPKSKTDLKAAVLEEWQKIPETYIQNLIVSMKRRLQAVCDANGSHTKY*

>AlternateORF-Long

MLSIKCFYSYGKLFCRSLLGDPVRFGPMRFGFSCVFDFSCHFLLQFITFCFNFMKNKMPGKRTTFEVAQLVYYNHQMGRQVSELADMFNLSKATIYNILNRANKEDRLEAKPVCGRPCKISDRDKRKILRKIEKNPQISLRDIAQEFKEESGVDVSHETVRKLLNSNDYTSRVARKKPLLSAANILKRLSFAQVHVNSSNDFWSNVIFCDESKMMLFYNDGPSRVWRKPLTALENRNIIPTVKFGKLSVMVWGCISSKGVGDLTFIENTMDARQYLSILQTHLVSSAQKFGFYEDNKPIFKFYQDNDPKHKAHMVRVWLLYNCGKVLDTPPQSPDMNPIENVWSYLKKKVAKRSPKSKTDLKAAVLEEWQKIPETYIQNLIVSMKRRLQAVCDANGSHTKY*

>AlternateORF-Short

MRFGFSCVFDFSCHFLLQFITFCFNFMKNKMPGKRTTFEVAQLVYYNHQMGRQVSELADMFNLSKATIYNILNRANKEDRLEAKPVCGRPCKISDRDKRKILRKIEKNPQISLRDIAQEFKEESGVDVSHETVRKLLNSNDYTSRVARKKPLLSAANILKRLSFAQVHVNSSNDFWSNVIFCDESKMMLFYNDGPSRVWRKPLTALENRNIIPTVKFGKLSVMVWGCISSKGVGDLTFIENTMDARQYLSILQTHLVSSAQKFGFYEDNKPIFKFYQDNDPKHKAHMVRVWLLYNCGKVLDTPPQSPDMNPIENVWSYLKKKVAKRSPKSKTDLKAAVLEEWQKIPETYIQNLIVSMKRRLQAVCDANGSHTKY*
